# *m*-AAA proteases, mitochondrial calcium homeostasis and neurodegeneration

**DOI:** 10.1038/cr.2018.17

**Published:** 2018-02-16

**Authors:** Maria Patron, Hans-Georg Sprenger, Thomas Langer

**Affiliations:** 1 Max Planck Institute for Biology of Aging, Cologne, Germany; 2 Cologne Excellence Cluster on Cellular Stress Response in Aging-Associated Disease (CECAD), and Center for Molecular Medicine Cologne (CMMC), University of Cologne, Cologne, Germany

**Keywords:** mitochondria, *m*-AAA proteases, AFG3L2, SPG7, spinocerebellar ataxia, hereditary spastic paraplegia, mitochondrial calcium uniporter

## Abstract

The function of mitochondria depends on ubiquitously expressed and evolutionary conserved *m*-AAA proteases in the inner membrane. These ATP-dependent peptidases form hexameric complexes built up of homologous subunits. AFG3L2 subunits assemble either into homo-oligomeric isoenzymes or with SPG7 (paraplegin) subunits into hetero-oligomeric proteolytic complexes. Mutations in *AFG3L2* are associated with dominant spinocerebellar ataxia (SCA28) characterized by the loss of Purkinje cells, whereas mutations in *SPG7* cause a recessive form of hereditary spastic paraplegia (HSP7) with motor neurons of the cortico-spinal tract being predominantly affected. Pleiotropic functions have been assigned to *m*-AAA proteases, which act as quality control and regulatory enzymes in mitochondria. Loss of *m*-AAA proteases affects mitochondrial protein synthesis and respiration and leads to mitochondrial fragmentation and deficiencies in the axonal transport of mitochondria. Moreover *m*-AAA proteases regulate the assembly of the mitochondrial calcium uniporter (MCU) complex. Impaired degradation of the MCU subunit EMRE in AFG3L2-deficient mitochondria results in the formation of deregulated MCU complexes, increased mitochondrial calcium uptake and increased vulnerability of neurons for calcium-induced cell death. A reduction of calcium influx into the cytosol of Purkinje cells rescues ataxia in an AFG3L2-deficient mouse model. In this review, we discuss the relationship between the *m*-AAA protease and mitochondrial calcium homeostasis and its relevance for neurodegeneration and describe a novel mouse model lacking MCU specifically in Purkinje cells. Our results pledge for a novel view on *m*-AAA proteases that integrates their pleiotropic functions in mitochondria to explain the pathogenesis of associated neurodegenerative disorders.

## Introduction

Mitochondria are dynamic, double-membrane-bound organelles that reside in all eukaryotic cells with a prominent role in metabolism, ion homeostasis and cell death. They exert essential anabolic and catabolic functions and produce ATP through oxidative phosphorylation, while mitochondrial outer membrane permeabilization, regulated by Bcl-2 family proteins, induces the intrinsic apoptotic pathway. ATP synthesis depends on the maintenance of a proton gradient across the mitochondrial inner membrane, which exhibits low permeability to ions. Still, the membrane harbors a variety of highly regulated, selective and non-selective ion channels, which preserve mitochondrial and cellular ion homeostasis.

**Figure 1:**
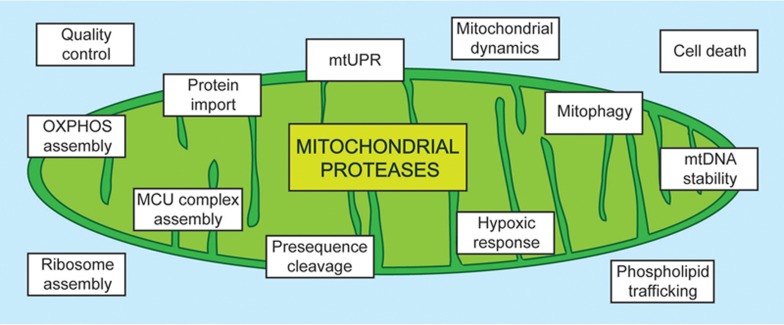
Functions of mitochondrial proteases. Mitochondrial proteases execute multiple functions that are crucial for mitochondrial function. Besides protein quality surveillance, many proteases control a variety of processes within mitochondria (shown in boxes) by either proteolytic processing or by mediating the rapid turnover of regulatory proteins.

Perturbations of mitochondrial functions are associated with ageing and the development of many pathological conditions, highlighting the central role of mitochondria for cell survival and tissue homeostasis. Numerous pathogenic mutations in nuclear-encoded mitochondrial proteins have been identified that often cause neuromuscular disorders. This is exemplified by proteases, which reside in different mitochondrial compartments and preserve the functional integrity of mitochondria^1,2^. They constitute an intra-organellar quality control system, which ensures the complete degradation of misfolded and damaged mitochondrial proteins to amino acids. Moreover, multiple processes in mitochondria are under the control of proteases, which mediate processing events and limit the stability of short-lived mitochondrial proteins (Figure 1). Thereby, proteases regulate the import of nuclear-encoded proteins into mitochondria, the stability of the mitochondrial genome and the synthesis of mitochondrially encoded subunits of the respiratory chain complexes. Similarly, the synthesis and intramitochondrial transport of phospholipids and the dynamic behavior of mitochondrial membranes that constantly fuse and divide are under proteolytic control. Considering the pleiotropic roles of proteases for the functional integrity of mitochondria, it is not surprising that a steadily increasing number of pathogenic mutations are being identified in genes encoding mitochondrial proteases (Table 1). However, the pathogenic mechanisms underlying the resulting diseases may vary significantly and to account for them, the contributions of the loss of regulatory functions in combination with an impaired mitochondrial quality control must be considered.

## AAA proteases — proteolytic machines in the mitochondrial inner membrane

AAA proteases constitute a family of conserved and ubiquitous ATP-dependent proteases that are embedded in the mitochondrial inner membrane. Subunits of these hexameric proteolytic complexes harbor an ATPase domain of the AAA family (ATPases associated with diverse cellular activities) and a metallopeptidase of the M48 family. AAA proteases exposing these catalytic domains to the matrix are termed *m*-AAA proteases in contrast to the *i*-AAA proteases, which exhibit an inverted topology in the inner membrane with catalytic domains protruding into the intermembrane space. An *i*-AAA protease is composed of YME1L subunits whereas various isoenzymes of mammalian *m*-AAA proteases have homologous subunits but differ in their subunit composition^3,4,5^. For example, AFG3L2 subunits can either form homo-oligomeric complexes or hetero-oligomerize with SPG7 (paraplegin) subunits that only assemble into hetero-oligomeric *m*-AAA proteases. Moreover, an additional *m*-AAA protease subunit, AFG3L1, is expressed in rodents^6^ and can either homo-oligomerize or assemble with AFG3L2 and SPG7 subunits^4^.

Studies on yeast homologues of *m*- and *i*-AAA proteases provided the first insight into the structural organization of mitochondrial AAA proteases^7^ and revealed principles of membrane protein degradation by these proteolytic machines^8,9,10,11,12^. The assembly of AAA proteases into hexameric ring complexes allows proteolysis to occur in a protected environment within a proteolytic chamber. The chaperone-like activity of AAA domains ensures the specific recognition of misfolded, solvent exposed domains of membrane proteins^8,9^. After binding to the outer surface of AAA proteases, substrate proteins are extracted from the membrane bilayer into the proteolytic chamber for degradation^9,10,13^. Substrate dislocation is mediated by coordinated ATP hydrolysis with the ring of ATPase domains and central pore loops directly binding to substrate proteins^10,12^. AAA proteases exhibit a degenerate substrate specificity and can mediate the complete degradation of proteins to peptides, but structural constraints in substrate proteins, such as tightly folded domains, may limit proteolysis and allow proteolytic processing^14^. This functional plasticity makes AAA proteases versatile proteolytic machines with multiple functions within mitochondria.

## AAA proteases and neurodegeneration

Mutations in genes encoding AAA protease subunits have been associated with neuronal loss and neurodegeneration in humans. Homozygous mutations in *YME1L* were found to cause a mitochondriopathy with optic atrophy in a consanguineous pedigree^15^ but it remains to be seen whether mutations in *YME1L* are of broader pathogenic relevance. Recessive mutations in *SPG7* coding for the *m*-AAA protease subunit paraplegin cause hereditary spastic paraplegia (HSP7)^16^. The main clinical features of this frequent mitochondriophathy are weakness and spasticity of the lower limbs, loss of vibratory sense and urinary urgency due to degeneration of motor axons of the cortico-spinal tracts. Manifestations of the disease can also include cortical and cerebellar atrophy, amyotrophy and mental retardation. Mutations in *AFG3L2* coding for the second *m*-AAA protease subunit are associated with spinocerebellar ataxia type 28 (SCA28), which is accompanied by the loss of Purkinje cells^17^. SCA28 is an autosomal dominantly inherited, rare ataxia with juvenile-onset, which is characterized by progressive gait and limb ataxia with eye movement abnormalities due to cerebellar abnormalities^18^.

The association of mutations in *SPG7* and *AFG3L2* with distinct neurodegenerative disorders can be explained by the formation of different isoenzymes by the two subunits: whereas the loss of SPG7 impairs specifically the formation of hetero-oligomeric *m*-AAA proteases, both homo- and hetero-oligomeric forms are affected in the absence of AFG3L2. As both isoenzymes appear to exert largely overlapping substrate specificities, the relative expression of *SPG7* and *AFG3L2* may contribute to the cell-type specificity in disease. This interpretation is consistent with the identification of recessive mutations in *AFG3L2* that affect the interaction of AFG3L2 with SPG7 as causative for spastic ataxia 5 (SPAX5). SPAX5 is a severe, early onset autosomal recessive ataxia, which is characterized by impaired ambulation, cerebellar ataxia and dystonia, thus combining clinical features of both HSP7 and SCA28^19^.

## Studying neuronal loss in *m*-AAA protease-deficient mouse models

The expression of *Spg7* and *Afg3l2* varies significantly between different tissues in mice, with a tenfold higher expression of *Afg3l2* and a fourfold higher expression of *Spg7* in the brain when compared to liver mitochondria^4^. Moreover, differences exist between the relative expression of *Spg7* and *Afg3l2* in the mouse brain, suggesting a prominent role for AFG3L2 in the brain and cooperative role for SPG7 in hippocampal neurons and Purkinje cells^20^. Different expression levels of *Spg7* and *Afg3l2* and the presence of homo- and hetero-oligomeric *m*-AAA proteases in different amounts may therefore contribute to the cell-type specificity in disease. Notably, the rodent-specific *m*-AAA protease subunit AFG3L1 is only expressed at low levels in the mouse brain. The loss of SPG7 or AFG3L2 in neurons therefore mimics the situation in humans, making mice a suitable model system to study the pathogenic mechanism of HSP7 and SCA28.

Mice lacking *Spg7* show mild and slowly progressive motor impairment associated with distal axonopathy of spinal and peripheral axons^21^. Mitochondrial morphological abnormalities occur in synaptic terminals and in distal regions of axons before axonal swelling and onset of motor impairment^21^. On the other hand, homozygous missense mutations in *Afg3l2* in a spontaneous mutant strain and the loss of AFG3L2 in *Afg3l2*^−/−^ mice cause a severe neuromuscular phenotype with hind limbs paresis, a severe defect in axonal development with delayed myelination and impairment of axonal radial growth, culminating in death shortly after birth. Mitochondrial morphology in these mice is abnormal with a strong impairment of the respiratory chain complexes resulting in reduced mitochondrial ATP production^22^. Thus, as in human disease, mutations in *Spg7* and *Afg3l2* cause distinct phenotypes in mice. *Afg3l2*-mutated mice show alterations in both the central nervous system (CNS) and peripheral nervous system with the cerebellum being the most strongly affected. The severe consequences of the loss of AFG3L2 can be explained by the inability of SPG7 to form *m*-AAA protease complexes in the absence of AFG3L2, whereas AFG3L2 is able to assemble into functional, homo-oligomeric complexes when SPG7 is absent. Notably, despite similar expression levels of *Spg7* and *Afg3l2* in murine astrocytes, oligodendrocytes and neurons, the latter are more susceptible to mutations in *Afg3l2*, likely reflecting differences in the metabolic profiles of these cell types^23^.

To delineate pathogenic mechanisms underlying neurodegeneration in AFG3L2 deficiency, functional studies have been performed in heterozygous *Afg3l2*^+/−^ mice. These are largely normal and do not show impaired axonal development but display a mild late-onset degenerative phenotype at least in certain experimental settings^24^. The progressive deficit in motor coordination in *Afg3l2*^+/−^ mice is the result of dark cell degeneration of Purkinje cells, characterized morphologically by cytoplasmic darkening, nuclear condensation and neuronal shrinkage. In these cells mitochondria appear swollen with disrupted cristae^24^ and show an impaired ability for trafficking and buffering cytosolic calcium (Ca^2+^) waves^25^. Neurological deficiencies are aggrevated when *Afg3l2*^+/−^ mice are bred with *Spg7*^−/−^ mice^20^. Such mice show loss of balance, tremor and ataxia, characterized by reactive astrogliosis and prominent cerebellar degeneration with loss of Purkinje cells and parallel fibers^20^. These phenotypes highlight the critical role of the expression levels of *m*-AAA protease subunits for neuronal survival.

## Delineating the pathogenic mechanisms of neurodegeneration in SCA28

Purkinje cells exclusively receive excitatory synapsis and, as the only efferent projections from the cortex, they modulate the activity of others neurons in the deep cerebellar nuclei (DCN). Accordingly, loss of these cells leads to hyper-excitable neurons of DCN with immediate consequence on the regulation of the fine movement^26^. Purkinje cells express high levels of AFG3L2 and SPG7 and are highly vulnerable towards the loss of the *m*-AAA protease in SCA28. Deletion of *Afg3l2* specifically in Purkinje cells triggers mitochondrial fragmentation and an altered distribution of mitochondria in the dendritic tree^27^. Accordingly, AFG3L2 is required for anterograde axonal transport of mitochondria in murine cortical neurons, suggesting that impaired mitochondrial trafficking leads to the progressive depletion of mitochondria from axons causing the late onset of the disease^28^.

Further studies in cultured cells have unraveled the mechanism of how the *m*-AAA protease influences mitochondrial morphology. Loss of the *m*-AAA protease activates the inner membrane peptidase OMA1^29^, which specifically cleaves the dynamin-like GTPase OPA1, a central component of the mitochondrial fusion machinery^30^. OMA1 limits the accumulation of fusion-active long OPA1 variants and leads to mitochondrial fragmentation due to ongoing fission events^29,30,31,32^. Misfolded, mitochondrial-encoded proteins accumulating in the absence of the *m*-AAA protease were found to activate OMA1 and to induce mitochondrial fragmentation^29,33,34^. The *m*-AAA protease is thus emerging as an important sensor coordinating the synthesis from mitochondrial and nuclear genomes. However, OMA1 activation upon loss of AFG3L2 and mitochondrial fragmentation apparently does not play a prominent role in axonal degeneration, as ablation of *Oma1* in AFG3L2-deficient neurons restores mitochondrial tubulation but not the axonal transport of mitochondria^28,29^. Thus, mitochondrial fragmentation appears to occur secondarily to other mitochondrial deficiencies.

AFG3L2-deficient Purkinje cells show respiratory defects and at early stages accumulate mitochondrial-encoded respiratory chain subunits at reduced levels^27^, defects that could explain the fragmentation of the mitochondrial network. The *m*-AAA protease is required for the synthesis of mitochondrial-encoded proteins in liver and brain mitochondria^27,35^ and regulates the assembly of mitochondrial ribosomes^27^. Studies in yeast have revealed that the *m*-AAA protease mediates the proteolytic processing of the ribosomal protein MrpL32, a prerequisite for mitochondrial translation^14,35^. While it remains to be demonstrated how the *m*-AAA protease regulates ribosome assembly and gene expression in mammalian mitochondria, it is conceivable that defective mitochondrial protein synthesis and respiratory defects are central pathogenic factors in AFG3L2-related neurodegeneration^27^. In agreement with this hypothesis, antioxidants or decreasing phospho-tau levels were found to rescue axonal trafficking defects in neurons lacking AFG3L2^28^, suggesting that reactive oxygen species signaling leads to cytoskeletal modifications that impair the axonal transport of mitochondria.

## Ca^2+^-dependent neuronal death and neurological diseases

These studies unraveled fundamental roles of *m*-AAA proteases for the functional integrity of mitochondria and neuronal survival, but the striking cell-type specificity of related neurodegenerative disorders remains unexplained. What are the features of the target cells that make them especially vulnerable to the loss of AFG3L2 or SPG7? Purkinje cells that are predominantly affected in ataxias are characterized by high-firing frequencies and express a variety of Ca^2+^ channels, Ca^2+^-sensitive phosphatases and kinases, and Ca^2+^-binding proteins to ensure efficient Ca^2+^ buffering^36^. Indeed, increasing evidence suggests that impaired cellular Ca^2+^ signaling plays a role in the etiology of various spinocerebellar ataxias^36,37^ (Table 2). Mitochondria can take up Ca^2+^ ions released from the endoplasmic reticulum, the major cellular Ca^2+^ store^38^. Being localized in close proximity to the plasma membrane, they also modulate extracellular Ca^2+^ influx, a mechanism that is specifically important in neurons^39^. They thus can act as a local buffer system determining the rate of Ca^2+^ waves through the cytosol. Ca^2+^ sequestration by mitochondria in the synapse can shape dynamic alterations in the cytosolic Ca^2+^ levels and strongly affect neurotransmitter release. Local alterations in the cytosolic Ca^2+^ concentration can position mitochondria to specific neuronal areas and the recruitment of mitochondria to specific areas changes the ion balance by controlling Ca^2+^ influx or release^40^.

Massive accumulation of Ca^2+^ ions in the matrix increases the permeability of the IMM by opening the mitochondria permeability transition pore (mPTP)^41^. Prolonged mPTP opening causes the collapse of the proton gradient, inhibition of ATP synthesis and dissipation of the ionic gradient across the IMM, which causes swelling, cristae unfolding and OMM rupture^42^. This mechanism also favors the release of pro-apoptotic proteins like cytochrome c. Deregulated Ca^2+^ homeostasis and mPTP opening are associated with many diseases of the CNS suggesting beneficial effects of an inhibition of the permeability transition mechanism^43^.

The first evidence for an important role of impaired Ca^2+^ homeostasis in SCA28 came from recent studies on heterozygous *Afg3l2*^+/−^ mice demonstrating that reducing the Ca^2+^ influx through the plasma membrane ameliorates motor deficits and dark cell degeneration of Purkinje cells^25^. In Purkinje cells, a transient increase in the cytosolic Ca^2+^ level can be induced by glutamate via a-amino-3-hydroxyl-5-methyl-4-isoxazole-propionate (AMPA) receptors or by the release of Ca^2+^ from the endoplasmic reticulum via metabotropic glutamate receptors (mGluR). Strikingly, reducing the dosage of mGluR1 by one half restores the motor performance of *Afg3l2*^+/−^ mice^25^. Similarly, when *Afg3l2*^+/−^ mice are fed with the antibiotic ceftriaxone promoting the clearance of glutamate from synapses, this suppresses the ataxic phenotype^45^, further substantiating the beneficial effects of reducing the cytosolic Ca^2+^ concentration in *AFG3L2*-deficient Purkinje cells.

These studies point to a prominent role of disturbed cellular Ca^2+^ homeostasis in the pathogenesis of SCA28. It has been suggested that mitochondrial fragmentation^46^ or mitochondrial depolarization^25^ impairs Ca^2+^ influx into AFG3L2-deficient mitochondria resulting in an increase of cytosolic Ca^2+^ concentrations and neuronal death. However, as mitochondrial Ca^2+^ buffering affects mainly local Ca^2+^ concentrations in the cytosol, alternative scenarios explaining the disturbed Ca^2+^ homeostasis in AFG3L2-deficient neurons must also be considered. Notably, recent work by Shanmughapriya *et al*. identified SPG7 amongst the proteins that co-immunoprecipitate with the well-known PTP regulator CyPD and suggested SPG7 to be an essential component of the mitochondrial PTP complex together with VDAC1^44^. Although such a function is difficult to reconcile with neuronal loss in HSP patients lacking SPG7, a possible regulatory function of SPG7 in the formation of PTP warrants further investigation.

## The neuronal interactome of *m*-AAA proteases

The striking cell-type specificity of *m*-AAA protease related diseases may reflect an increased dependence of Purkinje cells on AFG3L2 or the impaired proteolysis of neuron-specific substrates. The recent determination of the neuronal interactome of *m*-AAA proteases in mice provided the first insight into this question^47^. A point mutation in the Walker B motif of the ATPase domain of AFG3L2 inactivates the *m*-AAA protease but does not interfere with its assembly or with the binding of substrate proteins^12,29^. Importantly, this variant exerts a dominant negative effect on the activity of endogenous *m*-AAA proteases^29^ and its expression in the mouse forebrain therefore phenocopies the loss of AFG3L2 *in vivo* inducing atrophy and neuronal death^47^. Affinity purification of mutant *m*-AAA proteases from mouse brain led to the identification of the *m*-AAA protease-interacting protein 1 (MAIP1). MAIP1 is a ubiquitously expressed protein that, rather than being degraded, stably associates with *m*-AAA protease complexes. It contains a TIMM44-like domain, which is suggested to exert chaperone-like activity and which is also found in the mitochondrial protein translocase subunit TIMM44 and the mitochondrial ribosomal subunit MRPL45 (Mba1 in yeast). Interestingly, the α-proteobacterial AAA protease FtsH forms a complex with a related protein containing a TIMM44-like domain indicating that this complex may be an evolutionary ancestor of mitochondrial protein translocases^48^. MAIP1 is not required for the proteolytic activity of the *m*-AAA protease but it regulates the membrane insertion of the single span inner membrane protein EMRE, an essential subunit of the mitochondrial Ca^2+^ uniporter (MCU) complex^49^, linking the function of the *m*-AAA protease to mitochondrial ion homeostasis^47^. Further studies identified EMRE as a novel substrate of the *m*-AAA protease, which degrades non-assembled EMRE subunits and thereby ensures MCU assembly and mitochondrial Ca^2+^ homeostasis^47,50^.

## The *m*-AAA protease regulates MCU assembly and mitochondrial Ca^2+^ homeostasis

The MCU complex allows the regulated transport of Ca^2+^ ions across the inner membrane driven by the electrical gradient^51,52,53^. It consists of a pore-forming unit that includes MCU itself, a dominant negative variant MCUb^54^, a small protein EMRE^49^, and the MICU family that functionally shape the channel activity^55,56,57,58,59^. At resting conditions, MICU proteins act as a lid and prevent uncontrolled Ca^2+^ influx into the mitochondrial matrix, whereas they serve as positive activators when the Ca^2+^ concentration overcomes a specific threshold. This mechanism of regulation allows Ca^2+^ to fuel the Krebs cycle without inducing cell death. EMRE bridges MCU subunits with the C-terminal poly-aspartate tail of MICU1 (the main component of the MICU family)^57^. It is required for MCU channel activity^49,60^ but at the same time keeps the regulatory proteins of the MICU family bound to the pore-forming unit^61^. The analysis of MCU assembly revealed that EMRE, present at low levels, interacts with regulatory proteins of the MICU family before it assembles with MCU subunits into a functionally active MCU complex, illustrating the importance of a tight control of mitochondrial Ca^2+^ influx during MCU assembly^47^. Loss of MICU1 or disruption of the EMRE-MICU interaction leads to the dissociation of the regulatory proteins from the MCU complex, resulting in the accumulation of MCU-EMRE complexes that are constitutively open and allow accumulation of Ca^2+^ ions in the matrix^61,62,63^.

**Figure 2:**
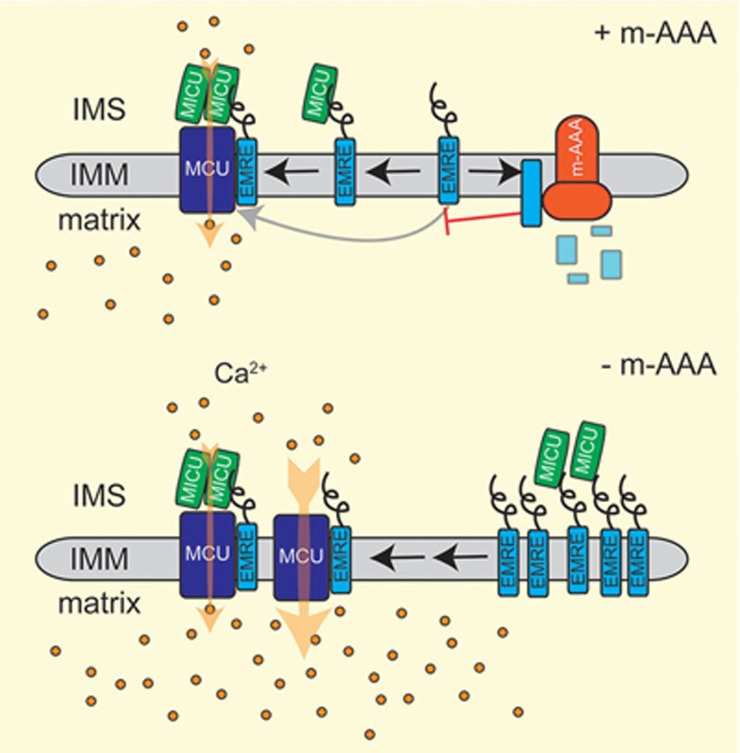
The *m*-AAA protease regulates MCU complex assembly by mediating the degradation of non-assembled EMRE subunits. An EMRE-MICU assembly intermediate binds to MCU subunits to form a gated MCU complex, while the *m*-AAA protease degrades non-assembled EMRE subunits. In the absence of the *m*-AAA protease, excess EMRE can bind either to MICU to form gated MCU complexes or can interact directly with MCU resulting in the formation of constitutively open MCU complexes lacking MICU regulatory proteins. This increases the vulnerability of cells to Ca^2+^ overload and Ca^2+^-induced cell death.

A similar effect on MCU assembly has been observed in *m*-AAA protease-deficient neurons. Degradation of non-assembled EMRE by the *m*-AAA protease ensures the binding of EMRE-MICU assembly intermediates with MCU subunits and the formation of regulated MCU complexes^47^. However, if EMRE proteolysis is impaired in the absence of the *m*-AAA protease, excess EMRE can bind to MCU independently of MICU regulatory proteins resulting in the accumulation of constitutively open MCU-EMRE complexes (Figure 2)^47,50^. These complexes also accumulate upon expression of an EMRE variant that is resistant to proteolysis by the *m*-AAA protease^50^, substantiating the critical role of EMRE degradation for MCU complex function. Of note, the relative expression of MCU, EMRE and MICU regulatory proteins in a cell will determine how impaired proteolysis of EMRE affects mitochondrial Ca^2+^ influx and cell survival, providing a rationale for the cell-type specificity in disease^64^.

## Does deregulated MCU assembly cause neurodegeneration in SCA28?

With AFG3L2 being responsible for the safety mechanism of degradation of unassembled EMRE, it is easy to envision that the accumulation of MCU-EMRE complexes and a massive mitochondrial Ca^2+^ influx will trigger Ca^2+^-induced death of *m*-AAA protease-deficient neurons^47^. Consistently, neuronal mitochondria lacking the *m*-AAA protease show an increased sensitivity to mitochondrial Ca^2+^ overload and opening of the mPTP^47^. This suggests that an excitotoxic increase of the mitochondrial Ca^2+^ concentration may induce Purkinje cell death in SCA28. The deleterious effect of the formation of ungated MCU complexes and massive mitochondrial Ca^2+^ influx on the survival of Purkinje cells is demonstrated by the ataxic phenotype of *Micu1*-deficient mice^62^. Notably, such a scenario may also explain why limiting the cytosolic Ca^2+^ accumulation suppresses ataxia in *Afg3l2^+/−^* mice^25^ and may open up new possibilities for therapeutic interventions using specific MCU inhibitors^65^.

**Figure 3:**
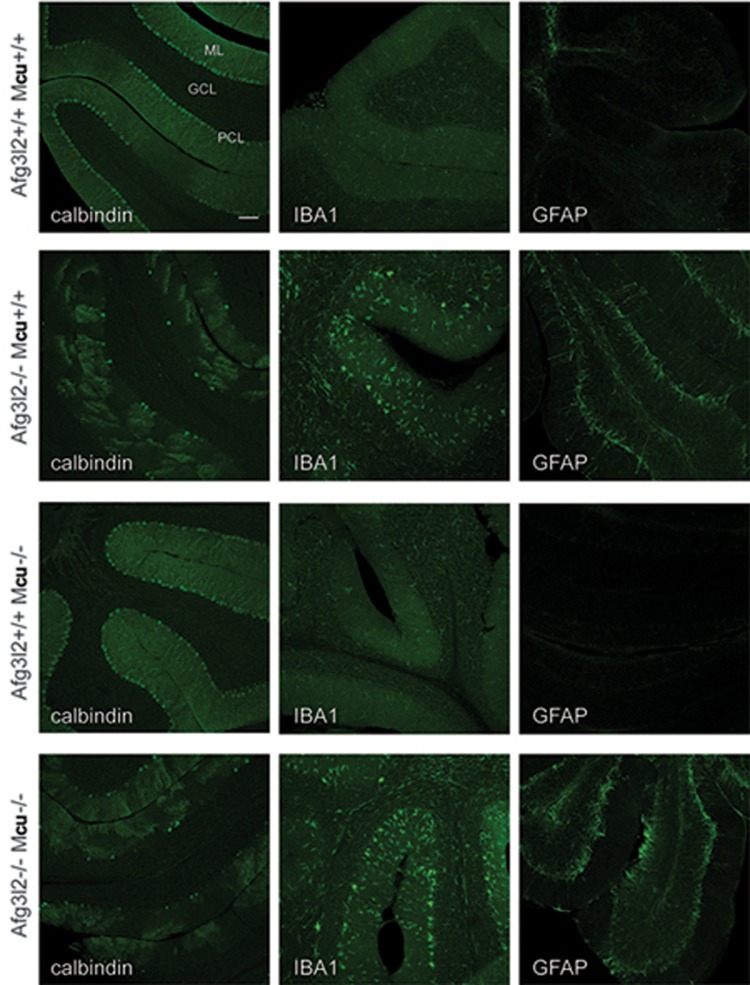
The deletion of *Mcu* does not prevent the degeneration of Purkinje cells lacking *AFG3L2*. *Afg3l2^fl/fl^* and *Mcu^fl/fl^* were bred with transgenic mice-expressing Cre recombinase under the control of the L7 promoter specifically in Purkinje cells^68^. Cerebelli of 6-week-old offsprings of the indicated phenotypes were stained with calbindin to visualize cerebellar Purkinje cells, IBA1 to mark activated microglia and GFAP for reactive astrocytes (as described in ref.^27^). The experiments are in agreement with the national ethical guidelines for studies in animals (84-02.04.2015.A402). GCL, granule cells layer; PCL, Purkinje cell layer; ML, molecular cell layer. A representative picture is shown for each condition. Scale bar, 100 μm.

To examine the relevance of mitochondrial Ca^2+^ overload for neuronal cell death, we recently deleted simultaneously *Mcu* and *Afg3l2* specifically in mouse Purkinje cells. To this purpose we crossed *Mcu^fl/fl^* (EMMA ID07445)^66,67^ or *Afg3l2^fl/fl^*^27^ mice with transgenic mice expressing Cre recombinase under the control of the L7 promoter, which drives the expression of the transgene between 2 and 3 weeks of age, i.e. when neurogenesis is completed^68^. The genetic background was shown to play an important role in the MCU phenotype with C57BL/6 whole-body knockout mice being lethal. In contrast, C57BL/6 mice lacking MCU specifically in Purkinje cells were born at the expected Mendelian ratio and did not exhibit any apparent phenotype. We observed a significant reduction of MCU protein level and Ca^2+^ uptake in isolated cerebellar mitochondria (data not shown). As MCU is considered to be the only pathway for the rapid uptake of Ca^2+^ into mitochondria, mitochondrial Ca^2+^ buffering appears to be dispensable for the survival of Purkinje cells. In contrast, mitochondrial Ca^2+^ overload in the absence of the MCU regulator MICU1 triggers death of Purkinje cells^62^. Notably, these findings also exclude impaired mitochondrial Ca^2+^ uptake in AFG3L2-deficient neuronal mitochondria as the pathogenic mechanism leading to neuronal loss in SCA28^25^. On the other hand, and as previously reported^27^, AFG3L2-deficient Purkinje cells degenerate starting from 6 weeks of age (Figure 3) and this is accompanied by an altered arborization and neuroinflammation (Figure 3). Strikingly, deletion of *Mcu* in Purkinje cells lacking AFG3L2 does not suppress neurodegeneration or the neuroinflammatory response, demonstrating that inhibiting mitochondrial Ca^2+^ uptake does not preserve the survival of Purkinje cells in this model (Figure 3). These results challenge the view that disturbed mitochondrial Ca^2+^ homeostasis and mitochondrial Ca^2+^ overload is solely responsible for neuronal death in SCA28. In view of the multiple functions of the *m*-AAA protease in mitochondria, it is conceivable that an impaired mitochondrial Ca^2+^ homeostasis in concert with other mitochondrial deficiencies triggers the loss of Purkinje cells in SCA28.

## Future perspectives

Recent years have seen major advances in our understanding of the role of *m*-AAA proteases in mitochondria and the pathogenesis of related neurodegenerative disorders. However, the pathogenic mechanisms linking mitochondrial defects in the absence of *m*-AAA proteases with neuronal loss and clinical manifestations of the diseases are still poorly understood. The identification of the MCU subunit EMRE as a substrate of the *m*-AAA protease, the regulatory role of EMRE proteolysis for MCU assembly and not least the protective effect of reduced cytosolic Ca^2+^ levels against ataxia in *Afg3l2^+/−^* mice all link disturbed mitochondrial Ca^2+^ homeostasis to SCA28. However, it is becoming evident that the pleiotropic functions of *m*-AAA proteases in mitochondria must be considered when analyzing disease pathogenesis. Future progress will therefore critically depend on the identification of further substrate proteins of AAA proteases. The characterization of the neuronal interactome of AFG3L2 may help in this way. These studies may also reveal whether functional differences between homo- and hetero-oligomeric *m*-AAA proteases exist and whether the striking cell-type specificity in HSP7 and SCA28 can be solely explained by tissue-specific differences in the expression of SPG7 and AFG3L2.

## Figures and Tables

**Table 1:** Diseases associated with loss of protease function

Gene	Disease	Inheritance	OMIM	References
*AFG3L2*	Spinocerebellar ataxia type 28 (SCA28); spastic ataxia-neuropathy syndrome	Dominant; recessive	610246; 614487	^17,19^
*CLPP*	Perrault syndrome 3	Recessive	614129	^69^
*HTRA2*	Parkinson disease type 13	Dominant	610297	^70,71,72^
*IMMP2L*	Gilles de la Tourette syndrome	Dominant	137580	^73^
*LONP1*	CODAS syndrome	Recessive	600373	^74^
*PARL*	Parkinson disease	Recessive	168600	^75^
*PMPCB*	Friedreich ataxia	Recessive	229300	^76,77^
*SPG7*	Hereditary spastic paraplegia 7 (HSP7)	Recessive	607259	^16,35^
*XPNPEP3*	Nephronophthisis-like nephropathy 1	Recessive	613159	^78^
*YME1L*	Optic atrophy 11	Recessive	617302	^15^

OMIM, Online mendelian inheritance in Man database; AFG3L2, AFG3-like protein 2; CLPP, ATP-dependent Clp protease proteolytic subunit; HTRA2, high-temperature requirement protein A2; IMMP2L, mitochondrial inner membrane protease 2; LONP1, lon protease homologue; PARL, presenilins-associated rhomboidlike protein; PMPCB, peptidase mitochondrial processing beta subunit; SPG7, paraplegin; XPNPEP3, X-Pro aminopeptidase 3; YME1L1, ATP-dependent zinc metalloprotease YME1L1.

**Table 2:** Role of Ca^2+^ in the etiology of SCA

Type of SCA	Protein	Effect on Ca^2+^ homeostasis	References
SCA1	Ataxin 1	Reduction of PC Ca^2+^-binding protein levels	^79,80^
SCA2	Ataxin 2	Increase Ca^2+^ release from ER stores	^81,82^
SCA3	Ataxin 3	Increase Ca^2+^ release from ER stores	^83^
SCA5	β-III-spectrin	Increase	^84^
SCA6	CACNA1A	Impaired Ca^2+^ flux into neurons	^85^
SCA14	PKCγ	Increase/Decrease	^86^
SCA15	ITPR1	Inositol 1,4,5-triphosphate Ca^2+^ signaling	^87^
SCA28	AFG3-like protein 2	MCU complex assembly	^47^

CACNA1A, Ca^2+^ voltage-gated channel subunit alpha1 A; PKCγ, protein kinase C subunit γ ITPR1, inositol 1,4,5-trisphosphate receptor type 1; SCA, spinocerebellar ataxias.

## Abbreviations

AMPA: a-amino-3-hydroxyl-5-methyl-4-isoxazole-propionate
CNS: central nervous system
DCN: deep cerebellar nuclei
GCL: granule cells layer
HSP: hereditary spastic paraplegia
IMM: inner mitochondrial membrane
IMS: intra membrane space
mGluR: metabotropic glutamate receptors
ML: molecular cell layer
PCL: Purkinje cell layer
PNS: peripheral nervous system
SCA: spinocerebellar ataxia
SPAX: spastic ataxia
